# Efeitos de curto prazo da pandemia de COVID-19 na realização de procedimentos de rastreamento, investigação diagnóstica e tratamento do câncer no Brasil: estudo descritivo, 2019-2020

**DOI:** 10.1590/S1679-49742022000100010

**Published:** 2022-03-07

**Authors:** Caroline Madalena Ribeiro, Flávia de Miranda Correa, Arn Migowski

**Affiliations:** 1 Instituto Nacional de Câncer José Alencar Gomes da Silva, Divisão de Detecção Precoce e Apoio à Organização de Rede, Rio de Janeiro, RJ, Brasil Instituto Nacional de Câncer José Alencar Gomes da Silva Divisão de Detecção Precoce e Apoio à Organização de Rede Rio de Janeiro RJ Brasil

**Keywords:** Detecção Precoce de Câncer, Técnicas e Procedimentos Diagnósticos, Terapêutica, Infecções por Coronavírus, Epidemiologia Descritiva, Detección Precoz del Cáncer, Técnicas y Procedimientos Diagnósticos, Terapéutica, Infecciones por Coronavirus, Epidemiología Descriptiva, Early Detection of Cancer, Diagnostic Techniques and Procedures, Therapeutics, Coronavirus Infections, Pandemics, Epidemiology, Descriptive

## Abstract

**Objetivo:**

Analisar efeitos de curto prazo da pandemia de COVID-19 no rastreamento, investigação diagnóstica e tratamento de câncer no Brasil.

**Métodos:**

Estudo descritivo, utilizando-se dados do Sistema de Informações Ambulatoriais e do Sistema de Informações Hospitalares, e Sistema de Informação do Câncer. Calculou-se a variação percentual mensal de procedimentos de rastreamento, diagnóstico e tratamento de câncer, em 2019 e 2020, além do tempo esperado para realização dos exames relacionados aos cânceres do colo do útero e de mama.

**Resultados:**

Em 2020, houve redução de 3.767.686 (-44,6%) exames citopatológicos, 1.624.056 (-42,6%) mamografias, 257.697 (-35,3%) biópsias, 25.172 cirurgias oncológicas (-15,7%) e 552 (-0,7%) procedimentos de radioterapia, comparando-se a 2019. Os intervalos de tempo para realização de exames de rastreamento de câncer do colo uterino e mama foram pouco afetados.

**Conclusão:**

Ações de controle do câncer foram afetadas pela pandemia, sendo necessárias estratégias para mitigar efeitos dos atrasos no diagnóstico e tratamento.

## Abstract (en)

**Objective:**

To analyze the short-term effects of the COVID-19 pandemic on cancer screening, diagnosis and treatment in Brazil.

**Methods:**

This was a descriptive study using data from the Outpatient and Hospital Information Systems, and the Cancer Information System. Monthly percentage variation of cancer screening, diagnosis and treatment procedures in 2019 and 2020 was calculated, as well as waiting time for cervical and breast cancer tests.

**Results:**

In 2020 cytopathology tests fell by 3,767,686 (-44.6%), screening mammograms fell by 1,624,056 (-42.6%), biopsies fell by 257,697 (-35.3%), cancer surgery fell by 25,172 (-15.7%), and radiotherapy procedures fell by 552 (-0.7%), compared to 2019. Time intervals for performing cervical and breast cancer screening exams were little affected.

**Conclusion:**

Cancer control actions were impacted by the pandemic, making it necessary to devise strategies to mitigate the effects of possible delays in diagnosis and treatment.

## Resumen

**Objetivo:**

Analizar los efectos a corto plazo de la pandemia por COVID-19 en rastreo, diagnóstico y tratamiento de cáncer en Brasil.

**Métodos:**

Estudio descriptivo utilizando datos del Sistemas de Información Ambulatoria y Hospitalaria del SUS y Sistema de Información del Cáncer. Se calculó la variación porcentual mensual en procedimientos de rastreo, diagnóstico y tratamiento de cáncer para 2019 y 2020, y el tiempo para realizar exámenes de cánceres de cérvix y mama.

**Resultados:**

En 2020 hubo reducción de 3.767.686 (-44,6%) en exámenes citopatológicos, 1.624.056 (-42,6%) en mamografías, 257.697 (-35,3%) en biopsias, 25.172 (-15,7%) en cirugías oncológicas y 552 (-0,7%) en radioterapia en comparación con 2019. Tiempos de los exámenes de rastreo para los cánceres de cérvix y de mama se vieron poco afectados.

**Conclusión:**

Acciones de control del cáncer se vieron impactadas por la pandemia, por lo que fue necesario diseñar estrategias para mitigar los efectos de posibles retrasos en diagnóstico y tratamiento.

## INTRODUÇÃO

**Figure:**
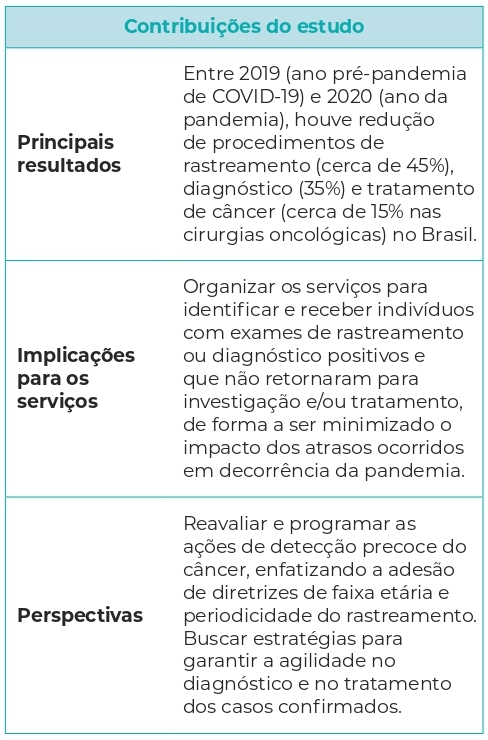

Atrasos no diagnóstico e tratamento do câncer podem ocorrer por diversos fatores, relacionados ao indivíduo atendido, aos profissionais e ao acesso e organização dos serviços de saúde, no âmbito do sistema de saúde.^1^^,^^2^ Entretanto, no ano de 2020, somou-se a esses fatores o efeito da pandemia causada pelo novo coronavírus.

No Brasil, o primeiro caso confirmado de COVID-19, doença causada pelo vírus SARS-CoV-2, ocorreu em fevereiro de 2020. Em março, alguns estados já apresentavam transmissão comunitária, levando à regulamentação dos critérios para isolamento e quarentena pelo Ministério da Saúde.^3^ Esses critérios foram aplicados de forma distinta, nos estados e municípios, considerando-se o perfil epidemiológico da doença em nível local^3^ e a organização político-administrativa do país. Em maio de 2021, pouco mais de um ano depois do primeiro caso confirmado, o Brasil acumulava mais de 18 milhões de casos da doença e 513 mil óbitos notificados.^4^

Todo o sistema de saúde foi impactado, não só pela demanda de atendimento dos casos de COVID-19, como também pelas medidas de isolamento e de distanciamento social que comprometeram o acesso dos indivíduos aos serviços de saúde.^5^

Em 2019, ocorreram cerca de 232 mil óbitos por câncer no país, sendo estimados 625 mil novos casos da doença para o ano de 2021.^6^ O diagnóstico precoce e o tratamento oportuno são indicados para todos os tipos de câncer, com o objetivo de aumentar a sobrevida e a qualidade de vida dos afetados.^1^^,^^2^

Embora não existam programas de rastreamento de câncer de base populacional no Brasil, há estratégias bem estabelecidas e diretrizes nacionais definidas especificamente para o rastreamento dos cânceres do colo do útero e de mama.^7^^,^^8^ Recomenda-se o rastreamento do câncer do colo do útero para mulheres de 25 a 64 anos, e do câncer de mama, para mulheres entre 50 e 69 anos.^7^^,^^8^

O Instituto Nacional de Câncer (INCA) recomendou, no início da pandemia, que exames de rastreamento poderiam ser adiados e que os casos com rastreamento positivo ou sintomáticos fossem investigados e, se confirmados, tratados.^9^ Posteriormente, considerando-se o cenário epidemiológico e a capacidade de resposta da rede de atenção à saúde no nível local, frente à pandemia, a retomada do rastreamento foi indicada, reforçando a priorização da confirmação diagnóstica e do tratamento.^10^

Recomendações sobre o cuidado de casos oncológicos durante a pandemia foram publicadas por sociedades médicas internacionais, encaminhando orientações sobre a prevenção da COVID-19, a necessidade de redução de visitas a enfermos internados e a utilização de estratégias para redução de circulação dos indivíduos afetados, como uso de telemedicina, fornecimento de medicamentos orais para ao menos três ciclos de tratamento e interrupção ou adiamento do tratamento, considerando-se riscos e benefícios individualmente.^11^^,^^12^

O objetivo do presente estudo foi analisar os efeitos de curto prazo da pandemia de COVID-19 no rastreamento, investigação diagnóstica e tratamento de câncer no Brasil. 

## MÉTODOS

Trata-se de estudo descritivo, com dados secundários sobre a realização de procedimentos de rastreamento, investigação diagnóstica e tratamento de câncer, no Brasil e suas macrorregiões, nos anos de 2019 e 2020. As fontes dos dados foram o Sistema de Informações Ambulatoriais (SIA/SUS) e o Sistema de Informações Hospitalares do Sistema Único de Saúde (SIH/SUS), as Autorizações de Procedimentos de Alta Complexidade (APAC) e o Sistema de Informação do Câncer (Siscan).

O diagnóstico e o tratamento do câncer envolvem diversos pontos da rede de atenção à saúde. Na atenção primária, profissionais de saúde identificam sinais e sintomas de alerta para o câncer e solicitam exames e procedimentos de investigação diagnóstica. No nível secundário da rede de atenção, indivíduos com sinais e sintomas suspeitos de câncer passam por consultas com especialistas e podem realizar biópsias e outros procedimentos de investigação diagnóstica. Os centros de apoio ao diagnóstico, especialmente laboratórios de histopatologia e clínicas radiológicas, são essenciais nessa etapa.

Casos confirmados de câncer são encaminhados às Unidades ou Centros de Tratamento de Alta Complexidade em Câncer (Unacon/Cacon), para realização de cirurgia, quimioterapia e radioterapia.

Para os cânceres do colo do útero e de mama, em que há recomendação de rastreamento, coleta do exame citopatológico e solicitação de mamografia, estes procedimentos são realizados no âmbito da atenção primária à saúde.

Para todos os tipos de cânceres registrados nos sistemas de informações do Sistema Único de Súde (SUS), no período de estudo, foram analisados os seguintes procedimentos: biópsias; exames histopatológicos; cirurgias oncológicas; quimioterapia; e radioterapia. Para os cânceres de mama e do colo do útero, especialmente, também foram considerados os procedimentos de rastreamento, exame citopatológico do colo do útero e mamografia.

Foram selecionadas as seguintes variáveis relacionadas aos procedimentos:

- rastreamento (exame citopatológico do colo do útero; mamografia de rastreamento);
- investigação diagnóstica (biópsia; exame anatomopatológico);
- tratamento (excisão do colo do útero de tipos 1, 2 e 3; cirurgia oncológica; quimioterapia; radioterapia);
- intervalos de tempo entre a realização de exames e a liberação dos laudos para rastreamento e diagnóstico dos cânceres do colo do útero e de mama (em dias: até 30; 31 a 60; mais de 60);
- adesão às diretrizes de população-alvo para o rastreamento - proporção de exames citopatológicos realizados em mulheres de 25 a 64 anos e proporção de mamografias de rastreamento realizadas em mulheres de 50 a 69 anos de idade.

Para se obter o quantitativo de procedimentos realizados, foram utilizadas bases de dados do SIA/SUS e do SIH/SUS, e das APAC, referentes aos meses de janeiro a dezembro, nos anos de 2019 e 2020. Realizou-se *download* das bases de dados mensais, disponíveis na página do Departamento de Informática do SUS (Datasus) na internet.^13^

As bases de dados dos meses de janeiro a março de 2021 foram incluídas no estudo para recuperação dos atendimentos realizados em outubro, novembro e dezembro de 2020 e faturados no ano seguinte, haja vista as regras do SUS permitirem o faturamento de procedimentos realizados na competência apresentada de até três meses anteriores.

Após *download* das bases de dados, os procedimentos de interesse foram selecionados utilizando-se o programa Tabwin, para se compor um novo de banco de dados.

Quanto às variáveis de rastreamento, as informações foram obtidas da base do SIA/SUS selecionando-se os procedimentos com códigos 02.03.01.001-9, 02.03.01.008-6 para câncer do colo do útero, e 02.04.03.018-8 para câncer de mama, previstos na tabela de procedimentos do SUS.^14^

Dados sobre investigação diagnóstica foram obtidos do SIA/SUS e incluíram os registros de biópsias, classificados pelos códigos 02.01.01.00-20 a 02.01.01.054-2, 02.01.01.066-6, 02.01.01.056-9, 02.01.01.058-5 e 02.01.01.060-7; e os registros de exames anatomopatológicos, classificados pelos códigos 02.03.02.003-0, 02.03.02.008-1, 02.03.02.002-2 e 02.03.02.007-3.^14^

As variáveis referentes ao tratamento foram obtidas no SIH/SUS e na APAC. Os procedimentos cirúrgicos incluídos foram as cirurgias oncológicas (subgrupo 0416) e as excisões do colo uterino (04.09.06.008-9, 04.09.06.003-8 e 04.09.06.030-5). Todos os procedimentos de quimioterapia e radioterapia registrados na base da APAC foram incluídos no estudo. 

Os intervalos de tempo entre a realização dos exames de rastreamento e investigação diagnóstica dos cânceres do colo do útero e de mama e a liberação de laudos foram obtidos na página eletrônica do Datasus, no Tabnet do Siscan, em março de 2021, a partir da variável ‘tempo do exame’.

Para a avaliação da adesão às diretrizes nacionais de rastreamento, utilizou-se o total de exames citopatológicos e mamografias realizados nas faixas etárias-alvo - 25-64 anos e 50-69 anos, respectivamente com o uso do Tabnet do Siscan, acessível -, na página do Datasus. 

Os dados foram analisados considerando-se o ano e o mês do atendimento. Na análise por ano, tomou-se o ano de 2019 como de pré-pandemia, e o ano de 2020 como período de pandemia. A comparação entre os registros mensais de cada ano permitiu identificar os meses de maior impacto da pandemia na realização de procedimentos. Apesar de os primeiros casos de COVID-19 terem ocorrido no Brasil em março de 2020, os dados de janeiro e fevereiro de 2019 e 2020 foram comparados para verificar se houve diferença na produção mensal dos procedimentos antes da pandemia.

 Dada a mudança na forma de registro do procedimento de radioterapia, ocorrida em maio de 2019, foram comparados, tão somente, os dados desse procedimento referentes ao período junho-dezembro de 2019 com os dados correspondentes do período junho-dezembro de 2020.^15^

Os intervalos de tempo entre a realização dos exames de rastreamento e a investigação diagnóstica foram calculados apenas para os cânceres do colo do útero e de mama, dada a disponibilidade de dados. Trata-se do intervalo de tempo, em dias, entre a coleta do exame citopatológico do colo do útero, a solicitação de mamografia, a coleta do material para biópsia de colo uterino ou de mama, e a liberação dos respectivos laudos no Siscan.

A análise comparativa das frequências absoluta e relativa (%) dos procedimentos realizados entre os períodos pré-pandemia e pandêmico foi realizada utilizando-se os recursos das planilhas Excel. O efeito de curto prazo da pandemia na realização de procedimentos foi avaliado por meio do cálculo das variações percentuais (VP), anual e mensal, nos procedimentos registrados em 2019 e 2020. A variação percentual mensal foi calculada pela diferença percentual entre os procedimentos realizados de janeiro a dezembro de 2020 e os procedimentos realizados de janeiro a dezembro de 2019.

Os intervalos de tempo entre a realização de exames e a liberação dos laudos para rastreamento e diagnóstico do câncer do colo do útero e do câncer de mama foram calculados pela diferença, em dias, entre a data de liberação do laudo e a data de coleta ou solicitação do exame. 

Os intervalos de tempo foram classificados da seguinte forma: até 30 dias, entre 31 e 60 dias e mais de 60 dias. Utilizou-se o teste qui-quadrado de Pearson para comparar as frequências das categorias, entres os anos de 2019 e 2020.

Para os exames histopatológicos de mama, calculou-se - separadamente - (i) o tempo de realização de exames para lesões identificadas no rastreamento (detecção da lesão por imagem) ou (ii) o tempo de realização de exames a partir de sinais de sintomas (detecção da lesão por exame clínico da mama), utilizando-se a variável ‘detecção da lesão’.

Para se verificar a adesão às diretrizes de detecção precoce, considerou-se a proporção de exames de rastreamento realizados nas populações-alvo: mulheres com idade entre 25 a 64 anos, para exame citopatológico do colo do útero; e mulheres com idade entre 50 e 69 anos, para mamografias de rastreamento.

O projeto do estudo não foi submetido à apreciação de um comitê de ética em pesquisa por utilizar exclusivamente dados secundários, de acesso público e sem a possibilidade de identificação dos indivíduos submetidos aos procedimentos de diagnóstico e tratamento oncológico analisados.

## RESULTADOS

Em 2020 (período de pandemia), houve redução de 3.767.686 (-44,6%) exames citopatológicos do colo do útero e de 1.624.056 (-42,6%) mamografias, em relação aos dados correspondentes de 2019 (período pré-pandemia). Observou-se que o mês de abril marcou o início da queda no registro dos exames, mais acentuada em maio de 2020, quando exames citopatológicos sofreram redução de 83,2% e mamografias de 83,4%, em relação a maio de 2019 (Tabela 1).

**Tabela 1:** - Variação percentual mensal e anual do número de procedimentos de rastreamento dos cânceres do colo do útero e de mama no âmbito do SUS,^a^ entre os períodos pré-pandemia (2019) e de pandemia da COVID-19 (2020), Brasil, 2019-2020

Mês de atendimento	Exames citopatológicos^b^				Mamografias de rastreamento		
	2019	2020	VP^c^		2019	2020	VP^c^
	N	N	%		N	N	%
Janeiro	654.127	632.873	-3,25		301.786	289.881	-3,94
Fevereiro	635.135	575.417	-9,40		307.995	291.771	-5,27
Março	662.877	640.703	-3,35		290.498	247.450	-14,82
Abril	711.117	323.568	-54,50		307.722	56.692	-81,58
Maio	727.215	121.980	-83,23		311.043	51.547	-83,43
Junho	663.913	113.838	-82,85		285.156	75.164	-73,64
Julho	710.996	165.169	-76,77		303.194	100.976	-66,70
Agosto	696.975	232.450	-66,65		304.087	127.807	-57,97
Setembro	698.288	324.850	-53,48		309.500	152.761	-50,64
Outubro	766.137	449.446	-41,34		403.624	254.484	-36,95
Novembro	791.763	571.541	-27,81		362.292	292.427	-19,28
Dezembro	730.194	529.216	-27,52		323.530	245.411	-24,15
Total	8.448.737	4.681.051	-44,59		3.810.427	2.186.371	-42,62

Fonte: Sistema de Informações Ambulatoriais do Sistema Único de Saúde (SIA/SUS); acesso em março de 2021.

a) SUS: Sistema Único de Saúde; b) O procedimento 02.03.01.001-9 (exame citopatológico cervicovaginal/microflora) foi incluído como procedimento de rastreamento, considerando-se a principal função do exame, e que o procedimento específico (02.03.01.008-6) é gerado apenas pelos serviços que implantaram o Sistema de Informação do Câncer (Siscan); c) VP: variação percentual.

Entre os procedimentos de investigação diagnóstica, houve redução de 257.697 (-35,3%) biópsias e de 737.852 (-26,7%) exames anatomopatológicos. Na análise mensal, abril de 2020 foi o mês de maior queda no registro de biópsias (-68,8%) e exames anatomopatológicos (-48,1%) (Tabela 2).

**Tabela 2:** - Variação percentual mensal e anual do número de procedimentos de investigação diagnóstica de câncer no âmbito do SUS,^a^ entre os períodos pré-pandemia (2019) e de pandemia da COVID-19 (2020), Brasil, 2019-2020

Mês de atendimento	Biópsias				Exames anatomopatológicos		
	2019	2020	VP^b^		2019	2020	VP^b^
	N	N	%		N	N	%
Janeiro	51.981	52.984	1,9		211.291	214.281	1,4
Fevereiro	57.867	53.849	-6,9		222.343	218.497	-1,7
Março	55.184	45.767	-17,1		211.759	210.267	-0,7
Abril	63.903	19.968	-68,8		234.397	121.565	-48,1
Maio	65.509	22.158	-66,2		203.368	108.525	-46,6
Junho	58.082	28.472	-51,0		220.218	124.201	-43,6
Julho	65.565	33.739	-48,5		245.496	145.923	-40,6
Agosto	65.859	38.106	-42,1		253.770	152.466	-39,9
Setembro	64.736	40.921	-36,8		243.585	169.052	-30,6
Outubro	66.092	45.955	-30,5		263.433	182.432	-30,7
Novembro	60.524	47.391	-21,7		230.912	190.875	-17,3
Dezembro	54.097	42.392	-21,6		222.110	186.746	-15,9
Total	729.399	471.702	-35,3		2.762.682	2.024.830	-26,7

Fonte: Sistema de Informações Ambulatoriais do Sistema Único de Saúde (SIA/SUS); acesso em março de 2021.

a) SUS: Sistema Único de Saúde; b) VP: variação percentual.

Considerando-se os procedimentos de tratamento do câncer no período analisado, houve redução de 8.689 (-32,6%) excisões do colo uterino, 25.172 (-15,7%) cirurgias oncológicas e 552 (-0,7%) procedimentos de radioterapia; e aumento de 117.631 (+3,2%) procedimentos de quimioterapia.

A queda no registro de excisões do colo uterino foi mais acentuada entre os meses de abril (-62,5%), e a de cirurgias oncológicas, entre os meses de maio, relativamente ao biênio 2019-2020 (-29,7%). Os procedimentos de quimioterapia e radioterapia não sofreram queda expressiva, no período analisado (Tabela 3).

**Tabela 3:** - Variação percentual mensal e anual de procedimentos de tratamento do câncer no âmbito do SUS,^a^ entre os períodos pré-pandemia (2019) e de pandemia da COVID-19 (2020), Brasil, 2019-2020

Mês de atendimento	Excisões do colo uterino				Cirurgias oncológicas^b^				Quimioterapia				Radioterapia^c^		
	2019	2020	VP^d^		2019	2020	VP^d^		2019	2020	VP^d^		2019	2020	VP^d^
	N	N	%		N	N	%		N	N	%		N	N	%
Janeiro	1.874	1.999	6,7		12.889	13.219	2,6		295.246	316.193	7,1		-	-	-
Fevereiro	2.155	1.899	-11,9		12.752	11.786	-7,6		297.113	313.074	5,4		-	-	-
Março	1.900	1.708	-10,1		12.014	12.792	6,5		297.173	316.169	6,4		-	-	-
Abril	2.323	870	-62,5		13.253	9.456	-28,7		296.840	306.629	3,3		-	-	-
Maio	2.366	1.111	-53,0		14.278	10.041	-29,7		303.183	308.810	1,9		-	-	-
Junho	2.145	1.251	-41,7		12.649	10.151	-19,7		302.173	311.164	3,0		12.581	11.464	8,9
Julho	2.203	1.387	-37,0		14.581	10.936	-25,0		306.241	314.376	2,7		11.824	12.269	-3,8
Agosto	2.488	1.542	-38,0		13.981	11.326	-19,0		309.181	318.442	3,0		11.702	11.469	2,0
Setembro	2.477	1.519	-38,7		14.265	11.694	-18,0		312.422	317.931	1,8		11.373	11.658	-2,5
Outubro	2.496	1.542	-38,2		14.747	11.816	-19,9		315.034	318.312	1,0		11.665	11.491	1,5
Novembro	2.189	1.683	-23,1		13.541	12.162	-10,2		313.191	320.043	2,2		10.887	10.825	0,6
Dezembro	2.056	1.472	-28,4		11.434	9.833	-14,0		313.509	317.794	1,4		10.583	10.887	-2,9
Total	26.672	17.983	-32,6		160.384	135.212	-15,7		3.661.306	3.778.937	3,2		80.615	80.063	-0,7

Fontes: Sistema de Informações Ambulatoriais do Sistema Único de Saúde (SIA/SUS) e Autorização de Procedimentos de Alta Complexidade (APAC); acesso em março de 2021.

a) SUS: Sistema Único de Saúde; b) Cirurgias oncológicas incluem somente os procedimentos realizados em hospitais habilitados na alta complexidade em oncologia, não contemplando cirurgias realizadas em hospitais gerais; c) Os dados do procedimento ‘radioterapia’ são referentes aos períodos junho-dezembro de 2019 e de 2020; d) VP: variação percentual.

No ano de 2020, a proporção de exames realizados no prazo de até 30 dias aumentou para todos os procedimentos de rastreamento e investigação diagnóstica dos cânceres do colo do útero e de mama no Brasil, exceto para mamografia de rastreamento (Tabela 4).

**Tabela 4:** - Distribuição dos intervalos de tempo entre a realização dos exames de rastreamento e investigação diagnóstica dos cânceres do colo do útero e de mama no âmbito do SUS,^a^ e a liberação do laudo, nos períodos pré-pandemia (2019) e de pandemia da COVID-19 (2020), Brasil, 2019-2020

Tipo de exame	Intervalo de tempo entre a realização do exame e a liberação do laudo													p-valor^b^
	2019							2020						
	Até 30 dias		31 a 60 dias		Mais de 60 dias			Até 30 dias		31 a 60 dias		Mais de 60 dias		
	N	%	N	%	N	%		N	%	N	%	N	%	
Exame citopatológico do colo do útero	3.273.340	46,0	2.593.930	36,4	1.250.546	17,6		1.913.613	47,7	1.346.164	33,6	747.977	18,7	<0,01
Mamografia de rastreamento	1.374.415	46,0	766.230	25,6	848.162	28,4		917.914	50,6	398.228	22,0	497.328	27,4	<0,01
Mamografia diagnóstica	38.382	48,3	18.827	23,7	22.204	28,0		25.867	49,5	10.991	21,0	15.377	29,4	<0,01
Histopatológico do colo do útero (biópsia)	24.655	58,7	10.209	24,3	7.118	17,0		17.572	64,5	4.750	17,4	4.921	18,1	<0,01
Histopatológico de mama - lesão palpável	10.467	54,3	3.954	20,5	4.855	25,2		8.765	58,2	3.024	20,1	3.268	21,7	<0,01
Histopatológico de mama - detecção por imagem	12.475	60,3	4.006	19,4	4.213	20,4		10.738	62,9	3.070	18,0	3.260	19,1	<0,01

Fonte: Sistema de Informação do Câncer (Siscan); acesso em março de 2021.

a) SUS: Sistema Único de Saúde; b) Teste qui-quadrado de Pearson.

Não houve variação nos tempos de realização do exame histopatológico, entre lesões detectadas pela identificação de sinais e sintomas e as oriundas do rastreamento mamográfico. Em 2019, 54,3% dos laudos de exames realizados por conta de alterações identificadas no exame clínico foram liberados em até 30 dias, enquanto para os exames provenientes do rastreamento, o percentual de laudos liberados dentro desse mesmo intervalo de tempo foi de 60,3%. Esse padrão foi mantido em 2020, com 58,2% para casos sintomáticos e 62,9% para casos oriundos do rastreamento (Tabela 4).

Os percentuais de exames realizados em mulheres nas faixas etárias-alvo do rastreamento no Brasil sofreram pouca variação, entre o período pré-pandemia e durante a pandemia, passando de 80,4% (2019) para 81,5% (2020) no rastreamento do câncer do colo do útero (25 a 64 anos), e de 64,8% (2019) para 64,4% (2020) no rastreamento do câncer de mama (50 a 69 anos).

A análise por macrorregiões nacionais revelou que no ano de 2020, período de pandemia, o Centro-Oeste foi a região a apresentar as maiores quedas nos procedimentos de rastreamento, em relação ao período pré-pandemia: -52,6% para exames citopatológicos e -46,5% para mamografias. Entre os procedimentos de diagnóstico, a biópsia teve maior queda na região Nordeste (-43,1%); e o exame anatomopatológico, maior queda na região Centro-Oeste (-41,9%). Nos procedimentos de tratamento, maiores quedas foram observadas nas cirurgias oncológicas (-21,3%) e nos tratamentos excisionais de lesões precursoras do câncer do colo do útero (-36,6%), ambas no cenário da região Nordeste; e na radioterapia (-8,0%), na região Norte. As regiões Nordeste, Sudeste e Centro-Oeste não apresentaram redução no número de registros de radioterapia em 2020 (Tabela 5).

**Tabela 5:** - Variação percentual (VP) de procedimentos de rastreamento, investigação diagnóstica e tratamento de câncer no âmbito do SUS,^a^ segundo macrorregiões nacionais, entre os períodos pré-pandemia (2019) e de pandemia da COVID-19 (2020), Brasil, 2019-2020

Procedimento	Norte				Nordeste				Centro-Oeste				Sudeste				Sul		
	2019	2020	VP		2019	2020	VP		2019	2020	VP		2019	2020	VP		2019	2020	VP
	N	N	%		N	N	%		N	N	%		N	N	%		N	N	%
Rastreamento																			
Exame citopatológico	521.566	264.348	-49,3		2.130.037	1.089.737	-48,8		517.272	245.265	-52,6		3.701.627	2.209.464	-40,3		1.578.235	872.237	-44,7
Mamografia de rastreamento	109.897	86.003	-21,7		865.870	476.306	-45,0		155.810	83.333	-46,5		1.909.072	1.099.763	-42,4		769.778	440.966	-42,7
Diagnóstico																			
Biópsia	28.129	18.306	-34,9		157.954	89.892	-43,1		35.257	22.995	-34,8		370.444	242.952	-34,4		137.615	97.557	-29,1
Exame anatomopatológico	121.259	91.932	-24,2		513.365	339.995	-33,8		176.312	102.357	-41,9		1.261.832	964.762	-23,5		689.914	525.784	-23,8
Tratamento																			
Excisão do colo do útero (tipos 1, 2 e 3)	2.393	1.534	-35,9		5.069	3.213	-36,6		1.412	904	-36,0		10.386	6.981	-32,8		7.412	5.351	-27,8
Cirurgia oncológica	4.835	4.318	-10,7		33.503	26.358	-21,3		9.863	8.945	-9,3		69.827	59.365	-15,0		42.356	36.226	-14,5
Quimioterapia	132.803	137.114	3,2		820.501	848.803	3,4		201.868	211.695	4,9		1.707.499	1.756.047	2,8		798.635	825.278	3,3
Radioterapia^b^	4.049	3.725	-8,0		18.648	18.798	0,8		3.851	4.363	13,3		36.422	36.712	0,8		17.645	16.465	-6,7

Fonte: Sistema de Informações Ambulatoriais do Sistema Único de Saúde (SIA/SUS); acesso em março de 2021.

a) SUS: Sistema Único de Saúde; b) Os dados do procedimento ‘radioterapia’ são referentes aos períodos junho-dezembro de 2019 e de 2020.

 Os meses de abril e maio de 2020 foram os mais críticos para a biópsia, que alcançou o patamar de queda de 78,5% em maio, na região Nordeste. Os exames anatomopatológicos, que incluem material proveniente de biópsias e cirurgias, tiveram maior queda em maio de 2020, na região Nordeste (-64,8%), e em junho de 2020, na região Centro-Oeste (-65,5%).

O tratamento de lesões precursoras do câncer do colo do útero teve as maiores quedas na região Nordeste, nos meses de abril (-84,6%) e junho de 2020 (-71,7%). As cirurgias oncológicas tiveram maior queda na região Nordeste, nos meses de maio (-56,5%) e junho (-36,8%). A maior queda na produção de radioterapia foi observada na região Sul, em junho (-18,7%). Comparado a 2019, o registro de procedimentos de quimioterapia manteve-se estável em 2020, em todas as regiões, inclusive com um pequeno aumento. Observou-se que, para todos os procedimentos e em todas as regiões, houve retomada na produção, principalmente a partir dos meses de agosto e setembro.

A proporção de exames histopatológicos do colo do útero e de mama realizados em até 30 dias foi maior em 2020, em praticamente todas as cinco grandes regiões do país, à exceção da região Norte, onde, para o histopatológico de mama, a proporção de exames realizados em mais de 60 dias passou de 8,4% em 2019 para 29,1% em 2020.

## DISCUSSÃO

No Brasil, no ano de 2020, quase todos os procedimentos relacionados ao rastreamento, investigação diagnóstica e tratamento de câncer sofreram queda na produção, em relação ao registrado em 2019; exceto a quimioterapia, que manteve o volume de produção, com discreto aumento em 2020. Exames de rastreamento sofreram as maiores reduções, especialmente nos meses de abril a junho de 2020.

As taxas de incidência da COVID-19, bem como as medidas restritivas adotadas pelos governos estaduais e locais, variaram entre estados e regiões. Contudo, observou-se um comportamento de queda na realização de procedimentos, em todo o país, e de forma mais acentuada nos meses de abril e maio, com retomada da produção no último trimestre de 2020.

Os resultados deste estudo indicaram importante redução na realização de procedimentos de rastreamento e diagnóstico de câncer no SUS, em todas as regiões do país, no contexto da pandemia de COVID-19, mesmo não tendo sido implementado *lockdown* no Brasil como um todo e tampouco adotadas medidas centralizadas de suspensão de atendimentos médicos eletivos. Além das restrições de mobilidade da população, que podem ter levado a uma redução na demanda por atendimentos ambulatoriais de rotina, houve sobrecarga dos serviços e profissionais de saúde.^16^

Considerando-se apenas os procedimentos de tratamento, aqueles específicos para lesões precursoras do câncer do colo do útero sofreram maior variação negativa. Diante deste achado, foram levantadas algumas hipóteses: (i) a possível opção das mulheres com diagnóstico de lesões precursoras por aguardarem o abrandamento da pandemia, para realização do tratamento; (ii) o impacto da sobrecarga dos serviços ambulatoriais para atendimento de demandas relacionadas à COVID-19; (iii) a suspensão de procedimentos eletivos; e (iv) a redução no rastreamento ter, como consequência, a diminuição dos diagnósticos de lesões precursoras.

Os intervalos de tempo entre a realização e a liberação dos laudos dos exames de rastreamento e investigação diagnóstica dos cânceres do colo do útero e de mama não sofreram variação expressiva. Foi possível observar redução nos tempos dos procedimentos de investigação diagnóstica em algumas regiões, indicando adesão às recomendações de priorizar casos sintomáticos e de investigação diagnóstica de pessoas com exames alterados no rastreamento durante a pandemia.^10^ Outra possibilidade de redução dos tempos totais reside na maior agilidade na liberação de laudos pelos laboratórios e clínicas radiológicas, devido à menor demanda de procedimentos eletivos e ao volume de exames realizados no período. Observou-se mudança de padrão no tempo de realização dos exames histopatológicos na região Norte, com aumento do tempo durante a pandemia, uma possível decorrência da implantação do Siscan em serviços de histopatologia do estado do Amazonas apenas em 2020, dificultando a comparação entre os anos de 2019 e 2020.^17^

No início da pandemia, houve orientação oficial enfática para a não realização de rastreamento fora das recomendações vigentes de faixa etária e periodicidade.^8^ Apesar disso, os dados demonstram que o padrão de falta de adesão persistiu em 2020,^18^ expondo os rastreados a maiores riscos, sem garantia da existência de benefícios.

O efeito da pandemia no cuidado dos indivíduos com câncer foi abordado em diversos estudos internacionais. Uma revisão sistemática, publicada em 2021, identificou 62 estudos realizados em 15 países, em sua maioria da Europa e América do Norte, relacionados a atrasos e interrupções no tratamento de pessoas com câncer como consequência da pandemia: atrasos no tratamento foram relatados por 77,5% dos indivíduos que responderam aos inquéritos objeto da pesquisa; uma taxa de interrupção do tratamento de 26,3% foi identificada nos estudos longitudinais; e uma redução de 30,0% nas internações relacionadas ao câncer.^19^ Não se encontraram estudos que avaliassem diretamente o efeito da pandemia no rastreamento e diagnóstico do câncer, embora haja estimativas realizadas por profissionais envolvidos na gestão de alguns programas de rastreamento de câncer em países de média e baixa renda.^20^

Estudo realizado em centros especializados em oncologia, localizados em 54 países, encontrou 88,0% a relatarem dificuldades no atendimento, durante a pandemia; e a perda de um ciclo de quimioterapia por mais de 10,0% dos indivíduos atendidos, reportada em 46,3% dos centros.^21^

Em estudo realizado no Reino Unido, estimou-se, por modelagem de base populacional, entre 3.291 e 3.621 mortes adicionais por câncer de mama, de esôfago, de pulmão e colorretal no período de cinco anos, como resultado dos atrasos no diagnóstico desses tipos de câncer atribuídos à pandemia.^22^

Nos Estados Unidos, estudo comparativo dos dados do Medicare, seguro nacional de saúde norte-americano, referentes ao período março-julho, entre os anos de 2019 e 2020, observou queda de 85,0% no rastreamento do câncer de mama no mês de abril, momento do pico da epidemia no país. O faturamento nos serviços de administração de quimioterapia também apresentou queda nos meses de abril a junho, variando entre 9,6 e 31,2%.^23^

Estudo realizado no Brasil identificou que as taxas médias de internação hospitalar para tratamento clínico de câncer diminuíram de 13,9 para 10,2 por 100 mil habitantes, entre 2019 e 2020, representando uma diferença de taxas de -3,7/100 mil hab. As admissões para tratamento oncológico cirúrgico mostraram um declínio da ordem de -5,8 por 100 mil hab., com diferenças regionais a variar entre -2,2 e -10,8 por 100 mil hab., e queda mais significativa nas regiões Sul e Sudeste.^24^

A comparação dos procedimentos realizados entre apenas dois anos, 2019 e 2020, pode ser vista como uma limitação do presente estudo, considerando-se que outros fatores também poderiam contribuir para a redução na oferta e utilização dos serviços. Entretanto, na análise mensal, é possível verificar que, nos meses anteriores à pandemia (janeiro e fevereiro), não houve quedas expressivas nos registros, observando-se, inclusive, aumento em alguns casos.

Devido à mudança na forma de registro e financiamento dos procedimentos de radioterapia, ocorrida em 2019,^15^ não foi possível avaliar sua variação entre março e maio, justamente os meses em que os demais procedimentos apresentaram maior variação na produção. Porém, a pequena variação ocorrida nos meses seguintes e o comportamento semelhante ao dos procedimentos de quimioterapia indicam que a radioterapia também sofreu menor efeito da pandemia. 

As cirurgias oncológicas avaliadas corresponderam às registradas apenas por hospitais habilitados em oncologia. É possível que as cirurgias realizadas em hospitais gerais tenham sofrido maior efeito da pandemia, por sobrecarga desses serviços e ocupação de leitos. Como não há um código específico para esse tipo de cirurgia realizada por hospitais não habilitados, não foi possível realizar essa análise neste estudo. 

Os resultados apresentados aqui, em concordância com os realizados em outros países, indicam que as ações de controle do câncer podem ter sofrido efeitos da pandemia, principalmente nos primeiros meses após o início da transmissão comunitária da doença.^21^^,^^23^ No Brasil, o rastreamento e o diagnóstico de câncer foram mais afetados que o tratamento, um resultado esperado considerando-se as recomendações vigentes e o balanço entre os riscos e os benefícios de manter ações de rastreamento em um cenário epidemiológico desfavorável como o da COVID-19.

Após um ano do início da pandemia, é fundamental o desenho de estratégias para amenizar danos dos possíveis atrasos resultantes da presença da COVID-19. Entre essas estratégias, cabe citar as seguintes iniciativas, incluídas no levantamento realizado pela Agência Internacional de Pesquisa em Câncer: (i) o desenvolvimento de aplicativos ou linhas de telefone específicas, para agendamento de consultas oncológicas e esclarecimento de dúvidas; (ii) os laudos de exames de rastreamento disponibilizados *online*; (iii) a teleconsulta para indivíduos com testes positivos; (iv) o transporte gratuito para indivíduos com teste de rastreamento positivo; e (v) o engajamento de jovens voluntários que identifiquem e apoiem indivíduos necessitados, com dificuldades de acesso ao atendimento oncológico.^20^

Apesar dos esforços já iniciados em diversos países, uma revisão sistemática para avaliação do impacto de medidas adotadas para reduzir os efeitos da COVID-19 no cuidado ao câncer, entre elas o adiamento ou alteração no esquema terapêutico, modificações dos intervalos de rastreamento e medidas de proteção nos centros de tratamento (alas isoladas para pessoas com COVID-19, teleconsultas), não identificou efeitos consistentes, dada a ausência de padronização nos métodos de avaliação, reiterando a importância de medir e documentar as estratégias adotadas.^25^

No período anterior à pandemia, o Brasil já apresentava dificuldades na organização do rastreamento, no acesso aos procedimentos diagnósticos e nos longos tempos de espera até o início do tratamento do câncer.^26^^-^^28^ Com o controle da pandemia, será necessário concentrar esforços na implementação de uma estratégia de priorização, baseada na estratificação do risco: confirmação diagnóstica e tratamento de casos sintomáticos suspeitos de câncer, e de pessoas com teste de rastreamento positivo, antes ou durante a pandemia; e busca ativa da população-alvo nunca rastreada ou em atraso de acordo com a periodicidade recomendada.^9^

Outros aspectos relevantes para o planejamento de ações relativas ao controle do câncer após o controle da pandemia são a realização de exames de rastreamento, na faixa etária e periodicidade adequadas, e a agilização no diagnóstico e tratamento dos casos confirmados. Apesar de este estudo não ter demonstrado variações nos registros de procedimentos por local de residência, em um país com dimensões continentais como é o Brasil, mostra-se fundamental reavaliar as condições de acesso e transporte de pessoas que vivem em áreas isoladas ou muito distantes dos centros de diagnóstico e tratamento, especialmente em um contexto de redução da circulação e mobilidade das pessoas.

Apesar dos efeitos devastadores da pandemia de COVID-19, o momento pode ser de planejamento, no sentido de oportunizar a reorganização da rede de atenção oncológica e dos programas de detecção precoce de câncer. É necessário evoluir do modelo de rastreamento oportunístico, em que exames são realizados por demanda própria do usuário ou solicitados pelo profissional de saúde, para o modelo organizado, em que a população-alvo é convidada a realizar exames na periodicidade recomendada. Dessa forma, poder-se-á estabelecer um controle centralizado das ações de rastreamento, estratégia fundamental para a maior efetividade das ações de controle do câncer no Brasil.^29^

## References

[1] Al-Azri MH (2016). Delay in cancer diagnosis: causes and possible solutions. Oman Med J.

[2] Hanna TP, King WD, Thibodeau S, Jalink M, Paulin GA, Harvey-Jones E (2020). Mortality due to cancer treatment delay: systematic review and meta-analysis. BMJ.

[3] Brasil. Ministério da Saúde (2020). Portaria no 356, de 11 de março de 2020. Dispõe sobre a regulamentação e operacionalização do disposto na Lei nº 13.979, de 6 de fevereiro de 2020, que estabelece as medidas para enfrentamento da emergência de saúde pública de importância internacional decorrente do coronavírus (COVID-19). Diário Oficial da União.

[4] Ministério da Saúde (BR) (2021). Coronavírus Brasil [Internet].

[5] Malta DC, Gomes CS, Silva AG, Cardoso LSM, Barros MBA, Lima MG (2021). Uso dos serviços de saúde e adesão ao distanciamento social por adultos com doenças crônicas na pandemia de COVID-19, Brasil, 2020. Ciênc Saúde Colet.

[6] Ministério da Saúde (BR). Instituto Nacional de Câncer José de Alencar Gomes da Silva (2015). Estimativa 2020: incidência de câncer no Brasil [Internet].

[7] Ministério da Saúde (BR). Instituto Nacional de Câncer José Alencar Gomes da Silva (2016). Diretrizes brasileiras para o rastreamento do câncer do colo do útero.

[8] Migowski A, Silva GA, Dias MBK, Diz MDPE, Sant’Ana DR, Nadanovsky P (2018). Guidelines for early detection of breast cancer in Brazil. II - New national recommendations, main evidence, and controversies. Cad Saude Publica.

[9] Ministério da Saúde (BR). Instituto Nacional de Câncer José Alencar Gomes da Silva (2021). Detecção precoce de câncer durante a pandemia de Covid-19 (Nota técnica . DIDEPRE/CONPREV/INCA - 30/3/2020) [Internet].

[10] Migowski A, Corrêa FM (2020). Recommendations for cancer early detection during covid-19 pandemic in 2021. Revista de APS.

[11] European Society for Medical Oncology (2021). Cancer patient management during the COVID-19 pandemic [Internet].

[12] American Society of Clinical Oncology (2020). COVID-19 guidance for practices translated to seven languages [Internet].

[13] Ministério da Saúde (BR) (2021). Datasus [Internet].

[14] Brasil. Ministério da Saúde (2007). Portaria GM no 2.848, de 06 de novembro de 2007. Aprova a tabela de procedimentos, medicamentos, órteses, próteses e materiais especiais - OPM do Sistema Único de Saúde. Diário Oficial da União.

[15] Brasil. Ministério da Saúde (2019). Portaria no 263, de 22 de fevereiro de 2019. Atualiza os procedimentos radioterápicos da Tabela de Procedimentos, Medicamentos, Órteses, Próteses e Materiais Especiais do Sistema Único de Saúde (SUS). Diário Oficial da União.

[16] Organização Pan-Americana da Saúde (2020). Informe de la evaluación rápida de la prestación de servicios para enfermedades no transmisibles durante la pandemia de COVID-19 en las Américas [Internet].

[17] Ministério da Saúde (BR). Instituto Nacional de Câncer José Alencar Gomes da Silva (2020). Informativo detecção precoce. Boletim 11.

[18] Migowski A, Dias MBK, Nadanovsky P, Silva GA, Sant’Ana DR, Stein AT (2018). Guidelines for early detection of breast cancer in Brazil. III - Challenges for implementation. Cad Saude Publica.

[19] Riera R, Bagattini AM, Pacheco RL, Pachito DV, Roitberg F, Ilbawi A (2021). delays and disruptions in cancer health care due to COVID-19 pandemic: systematic review. JCO Glob Oncol.

[20] Villain P, Carvalho AL, Lucas E, Mosquera I, Zhang L, Muwonge R (2021). Cross-sectional survey of the impact of the COVID-19 pandemic on cancer screening programs in selected low- and middle-income countries: Study from the IARC COVID-19 impact study group. Int J Cancer.

[21] Jazieh AR, Akbulut H, Curigliano G, Rogado A, Alsharm AA, Razis ED (2020). Impact of the COVID-19 pandemic on cancer care: a global collaborative study. JCO Glob Oncol.

[22] Maringe C, Spicer J, Morris M, Purushotham A, Nolte E, Sullivan R (2020). The impact of the COVID-19 pandemic on cancer deaths due to delays in diagnosis in England, UK: a national, population-based, modelling study. Lancet Oncol.

[23] Patt D, Gordan L, Diaz M, Okon T, Grady L, Harmison M (2020). Impact of covid-19 on cancer care: how the pandemic is delaying cancer diagnosis and treatment for american seniors. JCO Clin Cancer Inform.

[24] Costa AL, Ribeiro AL, Ribeiro AG, Gini A, Cabasag C, Reis RM (2021). Impact of COVID-19 pandemic on cancer-related hospitalizations in Brazil. Cancer Control.

[25] Pacheco RL, Martimbianco ALC, Roitberg F, Ilbawi A, Riera R (2021). Impact of strategies for mitigating delays and disruptions in cancer care due to COVID-19: systematic review. JCO Glob Oncol.

[26] Ribeiro CM, Dias MBK, Pla MAS, Correa FM, Russomano FB, Tomazelli JG (2019). Parameters for programming line of care procedures for cervical cancer in Brazil. Cad Saude Publica.

[27] Tomazelli JG, Silva GA (2017). Rastreamento do câncer de mama no Brasil: uma avaliação da oferta e utilização da rede assistencial do Sistema Único de Saúde no período 2010-2012. Epidemiol Serv Saúde.

[28] Costa RFA, Longatto-Filho A, Pinheiro C, Zeferino LC, Fregnani JH (2015). Historical analysis of the Brazilian cervical cancer screening program from 2006 to 2013: a time for reflection. PloS One.

[29] Basu P, Alhomoud S, Taghavi K, Carvalho AL, Lucas E, Baussano I (2021). Cancer screening in the coronavirus pandemic era: adjusting to a new situation. JCO Glob Oncol.

