# Supplementary material for: Short-term effects of the COVID-19 pandemic on cancer screening, diagnosis and treatment procedures in Brazil: a descriptive study, 2019-2020
Source: Epidemiol Serv Saude. 2022 Mar 7;31(1):e2021405. [Article in Portuguese] doi: 10.1590/S1679-49742022000100010 (PMC11346577; doi:10.1590/S1679-49742022000100010)
Supplement: Translation

# Translation: Short-term effects of the COVID-19 pandemic on cancer screening, diagnosis and treatment procedures in Brazil: a descriptive study, 2019-2020

**Authors:** Caroline Madalena Ribeiro, Flávia de Miranda Correa, Arn Migowski

**Affiliations:** 1 Instituto Nacional de Câncer José Alencar Gomes da Silva, Divisão de Detecção Precoce e Apoio à Organização de Rede, Rio de Janeiro, RJ, Brazil

**Keywords:** Early Detection of Cancer, Diagnostic Techniques and Procedures, Therapeutics, Coronavirus Infections, Pandemics, Epidemiology, Descriptive

## Abstract

**Objective:**

To analyze the short-term effects of the COVID-19 pandemic on cancer screening, diagnosis and treatment in Brazil.

**Methods:**

This was a descriptive study using data from the Outpatient and Hospital Information Systems, and the Cancer Information System. Monthly percentage variation of cancer screening, diagnosis and treatment procedures in 2019 and 2020 was calculated, as well as waiting time for cervical and breast cancer tests.

**Results:**

In 2020 cytopathology tests fell by 3,767,686 (-44.6%), screening mammograms fell by 1,624,056 (-42.6%), biopsies fell by 257,697 (-35.3%), cancer surgery fell by 25,172 (-15.7%), and radiotherapy procedures fell by 552 (-0.7%), compared to 2019. Time intervals for performing cervical and breast cancer screening exams were little affected.

**Conclusion:**

Cancer control actions were impacted by the pandemic, making it necessary to devise strategies to mitigate the effects of possible delays in diagnosis and treatment.

## INTRODUCTION

Delays in cancer diagnosis and treatment can occur due to several factors, related to the individual being treated, to health professionals and to service access and organization within the health system.^1^^,^^2^ However, in 2020, these factors were compounded by the effect of the pandemic caused by the novel coronavirus.

In Brazil, the first confirmed case of COVID-19, a disease caused by the SARS-CoV-2 virus, occurred in February 2020. By March, some states already had community transmission, leading to Ministry of Health regulation of criteria for isolation and quarantine.^3^ These criteria were applied differently in the states and municipalities, considering the epidemiological profile of the disease at the local level^3^ and the political-administrative organization of the country. In May 2021, just over a year after the first confirmed case, Brazil had accumulated more than 18 million reported COVID-19 cases and 513,000 reported deaths.^4^

The entire healthcare system was impacted, not only by the demand for care of COVID-19 cases, but also by the isolation and social distancing measures that compromised people’s access to healthcare services.^5^

In 2019, there were some 232,000 cancer deaths in Brazil, and 625,000 new cancer cases are estimated for the year 2021.^6^ Early diagnosis and timely treatment is indicated for all types of cancer, with the aim of increasing survival and quality of life of those affected.^1^^,^^2^

Although there are no population-based cancer screening programs in Brazil, there are well-established strategies and national guidelines defined specifically for cervical and breast cancer screening.^7^^,^^8^ Cervical cancer screening is recommended for women aged 25 to 64, and breast cancer screening for women aged 50 to 69.^7^^,^^8^

At the onset of the pandemic, the National Cancer Institute (Instituto Nacional de Câncer - INCA) recommended that screening tests should be postponed and that screen-positive or symptomatic cases should be investigated and treated if confirmed.^9^ Later on, considering the epidemiological scenario and the response capacity of the health care network at the local level in the face of the pandemic, the return of screening services was indicated, emphasizing prioritization of confirmation by diagnosis and treatment.^10^

Recommendations on care of cancer cases during the pandemic have been published by international medical societies, providing guidance on COVID-19 prevention, the need to reduce visits to hospitalized patients and the use of strategies to reduce the movement of affected individuals, such as using telemedicine, providing oral medications for at least three cycles of treatment and interrupting or postponing treatment, considering the risks and benefits on an individual basis.^11^^,^^12^

The objective of this study was to analyze the short-term effects of the COVID-19 pandemic on cancer screening, diagnosis and treatment in Brazil.

## METHODS

This is a descriptive study, using secondary data on the performance of cancer screening, diagnosis and treatment procedures in Brazil and its macro-regions in 2019 and 2020. The data sources were the Brazilian National Health Service (Sistema Único de Saúde - SUS) Outpatient Information System (Sistema de Informações Ambulatoriais - SIA/SUS) and the SUS Hospital Information System (Sistema de Informações Hospitalares - SIH/SUS), the High Complexity Procedure Authorizations database (Autorizações de Procedimentos de Alta Complexidade - APAC) and the Cancer Information System (Sistema de Informação do Câncer - SISCAN).

Cancer diagnosis and treatment involves several points in the health care network. In primary care, health professionals identify cancer warning signs and symptoms and refer cases for tests and diagnosis procedures. At the secondary level of the health care network, individuals with suspected cancer signs and symptoms are referred to specialists and may undergo biopsies and other diagnosis procedures. Diagnostic support centers, especially histopathology laboratories and radiology clinics, are essential at this stage.

Confirmed cancer cases are referred to High Complexity Cancer Treatment Units or Centers (Unidades/Centros de Tratamento de Alta Complexidade em Câncer - UNACON/CACON), for surgery, chemotherapy and radiotherapy.

When screening, cytology tests and mammography are recommended for cervical and breast cancer, these procedures are performed in primary health care services.

The following procedures were analyzed for all types of cancer registered on SUS information systems during the study period: biopsies; histopathology tests; cancer surgery; chemotherapy; and radiotherapy. Specifically in the case of breast and cervical cancer, screening, cervical cytology tests and mammography procedures were also analyzed.

The following variables were selected in relation to the procedures:

- screening (cervical cytology test; screening mammography);
- diagnosis (biopsy; anatomical pathology);
- treatment (type 1, 2 and 3 cervical excision; cancer surgery; chemotherapy; radiotherapy);
- time intervals between cervical and breast cancer screening and diagnosis testing and results (in days: up to 30; 31-60; more than 60);
- adherence to target population screening guidelines - proportion of cytology tests performed for women aged 25-64 and proportion of mammography screening performed for women aged 50-69.

We used the SIA/SUS, SIH/SUS and APAC databases for January to December 2019 and January to December 2020 in order to obtain the number of procedures performed. Therefore, we downloaded the monthly databases, available on the SUS Information Technology Department (DATASUS) website.^13^

The January to March 2021 databases were included in the study in order to include services performed in October, November and December 2020, but billed in the 2021, given that SUS rules allow billing of procedures performed in the previous three months.

After downloading the databases, the procedures of interest were selected using the Tabwin program, in order to compile a new database.

With regard to the screening variables, this information was obtained from the SIA/SUS database by selecting procedures recorded under codes 02.03.01.001-9 and 02.03.01.008-6 for cervical cancer, and code 02.04.03.018-8 for breast cancer, as per the SUS procedure list.^14^

Data on diagnosis were obtained from SIA/SUS and included biopsy records, classified by codes 02.01.01.00-20 to 02.01.01.054-2, 02.01.01.066-6, 02.01.01.056-9, 02.01.01.058-5 and 02.01.01.060-7; and anatomical pathology test records, classified by codes 02.03.02.003-0, 02.03.02.008-1, 02.03.02.002-2 and 02.03.02.007-3.^14^

The variables relating to treatment were obtained from SIH/SUS and the APAC database. The surgical procedures included were cancer surgery (subgroup 0416) and cervical excisions (04.09.06.008-9, 04.09.06.003-8 and 04.09.06.030-5). All chemotherapy and radiotherapy procedures recorded on the APAC database were included in the study.

The time intervals between performance of screening tests and diagnosis of cervical and breast cancer and the reports being made available were obtained via SISCAN Tabnet on the DATASUS webpage in March 2021, taking the ‘test time’ variable.

In order to assess adherence to national screening guidelines, we took the total number of cytology tests and mammograms performed in the target age groups - 25-64 years and 50-69 years, respectively, using SISCAN Tabnet available on the DATASUS webpage.

The data were analyzed considering the year and month of service provision. In the analysis by year, 2019 was taken as the pre-pandemic period, while 2020 was taken as the pandemic period. Comparison between the monthly records of each year allowed us to identify the months of greatest impact of the pandemic on the performance of procedures. Although the first COVID-19 cases occurred in Brazil in March 2020, data from January and February 2019 and 2020 were compared to see if there was a difference in monthly performance of procedures before the pandemic.

 Given the change in May 2019 the way the radiotherapy procedure was recorded, only data on this procedure for the period June-December 2019 were compared with the corresponding data for the period June-December 2020.^15^

The time intervals between screening tests and diagnosis were only calculated for cervical and breast cancer, given data availability. It is the time interval, in days, between collection of the cervical cytology test sample, data of referral for mammography, collection of samples for cervical or breast biopsy, and the release of the respective reports on SISCAN.

Comparative analysis of absolute and relative frequencies (%) of procedures carried out between the pre-pandemic and pandemic periods was performed using Excel spreadsheet resources. The short-term effect of the pandemic on the performance of procedures was evaluated by calculating annual and monthly percentage variation (PV) in procedures recorded in 2019 and 2020. Monthly percentage variation was calculated by the percentage difference between the procedures performed from January to December 2020, and the procedures performed from January to December 2019.

The time intervals between the performance of tests and the release of the reports for cervical cancer and breast cancer screening and diagnosis were calculated by the difference, in days, between the date of release of the report and the date of sample collection or request for tests. The time intervals were classified as follows: up to 30 days, between 31 and 60 days, and more than 60 days. Pearson’s chi-square test was used to compare the frequencies of the categories between the years 2019 and 2020.

For the breast cancer histopathology tests, we calculated - separately - (i) the time taken to test for lesions identified at screening (lesion detection by imaging) or (ii) the time taken to test for signs of symptoms (lesion detection by clinical breast examination), using the ‘lesion detection’ variable.

To verify adherence to early detection guidelines, we considered the proportion of screening tests performed in the target populations: women aged 25-64 years, for cervical cytology testing; and women aged 50-69 years, for mammography screening.

The study project was not submitted to a research ethics committee because it only used public-access secondary data, which did not identify individuals submitted to the cancer diagnosis and treatment procedures analyzed.

## RESULTS

In 2020 (pandemic period), cervical cytology tests fell by 3,767,686 (-44.6%) and mammograms fell by 1,624,056 (-42.6%), compared to the corresponding data for 2019 (pre-pandemic period). We found that April 2020 marked the beginning of the decline in test records. This was most pronounced in May 2020, when cytology tests reduced by 83.2% and mammograms by 83.4%, compared to May 2019 (Table 1).

**Table 1:** - Monthly and annual percentage variation in the number of SUS^a^ cervical and breast cancer screening procedures, between the pre-COVID-19 pandemic period (2019) and the COVID-19 pandemic period (2020), Brazil, 2019-2020

Month service provided	Cytology tests^b^				Mammography screening		
	2019	2020	PV^c^		2019	2020	PV^c^
	N	N	%		N	N	%
January	654,127	632,873	-3.25		301,786	289,881	-3.94
February	635,135	575,417	-9.40		307,995	291,771	-5.27
March	662,877	640,703	-3.35		290,498	247,450	-14.82
April	711,117	323,568	-54.50		307,722	56,692	-81.58
May	727,215	121,980	-83.23		311,043	51,547	-83.43
June	663,913	113,838	-82.85		285,156	75,164	-73.64
July	710,996	165,169	-76.77		303,194	100,976	-66.70
August	696,975	232,450	-66.65		304,087	127,807	-57.97
September	698,288	324,850	-53.48		309,500	152,761	-50.64
October	766,137	449,446	-41.34		403,624	254,484	-36.95
November	791,763	571,541	-27.81		362,292	292,427	-19.28
December	730,194	529,216	-27.52		323,530	245,411	-24.15
Total	8,448,737	4,681,051	-44.59		3,810,427	2,186,371	-42.62

Source: Brazilian National Health System Outpatient Information System (SIA/SUS); access in March 2021.

a) SUS: Brazilian National Health System; b) Procedure 02.03.01.001-9 (cervical-vaginal/microflora cytology test) was included as a screening procedure, considering the main purpose of the test, and that the specific procedure (02.03.01.008-6) is only generated by services that have implanted the Cancer Information System (SISCAN); c) PV: percentage variation.

Among the diagnostic procedures, biopsies fell by 257,697 (-35.3%) and anatomical pathology tests fell by 737,852 (-26.7%). In the monthly analysis, April 2020 was the month with the biggest drop in biopsy records (-68.8%) and anatomical pathology test records (-48.1%) (Table 2).

**Table 2:** - Monthly and annual percentage variation in the number of SUS^a^ cancer diagnosis procedures, between the pre-COVID-19 pandemic period (2019) and the COVID-19 pandemic period (2020), Brazil, 2019-2020

Month service provided	Biopsies				Anatomical pathology tests		
	2019	2020	PV^b^		2019	2020	PV^b^
	N	N	%		N	N	%
January	51,981	52,984	1.9		211,291	214,281	1.4
February	57,867	53,849	-6.9		222,343	218,497	-1.7
March	55,184	45,767	-17.1		211,759	210,267	-0.7
April	63,903	19,968	-68.8		234,397	121,565	-48.1
May	65,509	22,158	-66.2		203,368	108,525	-46.6
June	58,082	28,472	-51.0		220,218	124,201	-43.6
July	65,565	33,739	-48.5		245,496	145,923	-40.6
August	65,859	38,106	-42.1		253,770	152,466	-39.9
September	64,736	40,921	-36.8		243,585	169,052	-30.6
October	66,092	45,955	-30.5		263,433	182,432	-30.7
November	60,524	47,391	-21.7		230,912	190,875	-17.3
December	54,097	42,392	-21.6		222,110	186,746	-15.9
Total	729,399	471,702	-35.3		2,762,682	2,024,830	-26.7

Source: Brazilian National Health System Outpatient Information System (SIA/SUS); access in March 2021.

a) SUS: Brazilian National Health System; b) PV: percentage variation.

Considering cancer treatment procedures in the period analyzed, there was an 8,689 (-32.6%) reduction in cervical excisions, 25,172 (-15.7%) in cancer surgery and 552 (-0.7%) in radiotherapy procedures; and an increase of 117,631 (+3.2%) in chemotherapy procedures. The decline in cervical excision records was most pronounced when comparing April 2019 and April 2020 (-62.5%), and that of cancer surgery when comparing May 2019 and May 2020 (-29.7%).

There was no significant drop in chemotherapy and radiotherapy procedures during the period analyzed (Table 3).

**Table 3:** - Monthly and annual percentage variation in SUS^a^ cancer treatment procedures, between the pre-COVID-19 pandemic period (2019) and the COVID-19 pandemic period (2020), Brazil, 2019-2020

Month service provided	Cervical excision				Cancer surgery^b^				Chemotherapy				Radiotherapy^c^		
	2019	2020	PV^d^		2019	2020	PV^d^		2019	2020	PV^d^		2019	2020	PV^d^
	N	N	%		N	N	%		N	N	%		N	N	%
January	1,874	1,999	6.7		12,889	13,219	2.6		295,246	316,193	7.1		-	-	-
February	2,155	1,899	-11.9		12,752	11,786	-7.6		297,113	313,074	5.4		-	-	-
March	1,900	1,708	-10.1		12,014	12,792	6.5		297,173	316,169	6.4		-	-	-
April	2,323	870	-62.5		13,253	9,456	-28.7		296,840	306,629	3.3		-	-	-
May	2,366	1,111	-53.0		14,278	10,041	-29.7		303,183	308,810	1.9		-	-	-
June	2,145	1,251	-41.7		12,649	10,151	-19.7		302,173	311,164	3.0		12,581	11,464	8.9
July	2,203	1,387	-37.0		14,581	10,936	-25.0		306,241	314,376	2.7		11,824	12,269	-3.8
August	2,488	1,542	-38.0		13,981	11,326	-19.0		309,181	318,442	3.0		11,702	11,469	2.0
September	2,477	1,519	-38.7		14,265	11,694	-18.0		312,422	317,931	1.8		11,373	11,658	-2.5
October	2,496	1,542	-38.2		14,747	11,816	-19.9		315,034	318,312	1.0		11,665	11,491	1.5
November	2,189	1,683	-23.1		13,541	12,162	-10.2		313,191	320,043	2.2		10,887	10,825	0.6
December	2,056	1,472	-28.4		11,434	9,833	-14.0		313,509	317,794	1.4		10,583	10,887	-2.9
Total	26,672	17,983	-32.6		160,384	135,212	-15.7		3,661,306	3,778,937	3.2		80,615	80,063	-0.7

Sources: Brazilian National Health System Outpatient Information System (SIA/SUS) and High Complexity Procedure Authorizations (APAC); access in March 2021.

a) SUS: Brazilian National Health System; b) Cancer surgery only includes procedures performed in hospitals accredited for high complexity oncology, and does not include surgery performed in general hospitals; c) ‘Radiotherapy’ procedure data refer to June-December 2019 and June-December 2020; d) PV: percentage variation.

In 2020, the proportion of tests performed within up to 30 days increased for all cervical and breast cancer screening and diagnosis procedures in Brazil, except for screening mammography (Table 4).

**Table 4:** - Distribution of time intervals between screening tests and diagnosis of cervical and breast cancer in SUS^a^ services, and availability of results, in the pre-COVID-19 pandemic period (2019) and the COVID-19 pandemic period (2020), Brazil, 2019-2020

Type of examination	Time interval between examination and results													p-value^b^
	2019							2020						
	Up to 30 days		31-60 days		More than 60 days			Up to 30 days		31-60 days		More than 60 days		
	N	%	N	%	N	%		N	%	N	%	N	%	
Cervical cytology test	3,273,340	46.0	2,593,930	36.4	1,250,546	17.6		1,913,613	47.7	1,346,164	33.6	747,977	18.7	<0.01
Screening mammography	1,374,415	46.0	766,230	25.6	848,162	28.4		917,914	50.6	398,228	22.0	497,328	27.4	<0.01
Diagnostic mammography	38,382	48.3	18,827	23.7	22,204	28.0		25,867	49.5	10,991	21.0	15,377	29.4	<0,01
Cervical histopathology (biopsy)	24,655	58.7	10,209	24.3	7,118	17.0		17,572	64.5	4,750	17.4	4,921	18.1	<0.01
Breast histopathology - palpable lesion	10,467	54.3	3,954	20.5	4,855	25.2		8,765	58.2	3,024	20.1	3,268	21.7	<0.01
Breast histopathology - imaging detection	12,475	60.3	4,006	19.4	4,213	20.4		10,738	62.9	3,070	18.0	3,260	19.1	<0.01

Source: Cancer Information System (SISCAN); access in March 2021.

a) SUS: Brazilian National Health System; b) Pearson’s chi-square test.

There was no variation in the histopathology test time between lesions detected by identification of signs and symptoms and lesions identified by mammography screening. In 2019, 54.3% of reports on tests performed due to alterations identified in clinical examinations were released in up to 30 days, while for tests performed as a result of screening, 60.3% of reports were released within this same time interval. This pattern was maintained in 2020, with 58.2% for symptomatic cases and 62.9% for screening cases (Table 4).

The percentage of tests performed in women in the target age groups for screening in Brazil varied little between the pre-pandemic period and the pandemic period, from 80.4% (2019) to 81.5% (2020) for cervical cancer screening (25-64 years), and from 64.8% (2019) to 64.4% (2020) for breast cancer screening (50-69 years).

Analysis by national macro-regions revealed that in 2020, the pandemic period, the Midwest region had the biggest drops in screening procedures, compared to the pre-pandemic period: -52.6% for cytology tests and -46.5% for mammograms. Among the diagnosis procedures, the biggest drop in biopsies occurred in the Northeast (-43.1%); while the biggest drop in anatomical pathology tests occurred in the Midwest (-41.9%). With regard to treatment procedures, the biggest drops were seen in cancer surgery (-21.3%) and treatment with excision of cervical cancer precursor lesions (-36.6%), both in the Northeast; and radiotherapy (-8.0%) in the North. The Northeast, Southeast and Midwest regions showed no reduction in the number of radiotherapy records in 2020 (Table 5).

**Table 5:** - Percentage variation (PV) in cancer screening, diagnosis and treatment procedures in SUS^a^ services, by national macro-regions, between the pre-COVID-19 pandemic period (2019) and the COVID-19 pandemic period (2020), Brazil, 2019-2020

Procedure	North				Northeast				Midwest				Southeast				South		
	2019	2020	PV		2019	2020	PV		2019	2020	PV		2019	2020	PV		2019	2020	PV
	N	N	%		N	N	%		N	N	%		N	N	%		N	N	%
Screening																			
Cytology test	521,566	264,348	-49.3		2,130,037	1,089,737	-48.8		517,272	245,265	-52.6		3,701,627	2,209,464	-40.3		1,578,235	872,237	-44.7
Screening mammography	109,897	86,003	-21.7		865,870	476,306	-45.0		155,810	83,333	-46.5		1,909,072	1,099,763	-42.4		769,778	440,966	-42.7
Diagnosis																			
Biopsy	28,129	18,306	-34.9		157,954	89,892	-43.1		35,257	22,995	-34.8		370,444	242,952	-34.4		137,615	97,557	-29.1
Anatomical pathology test	121,259	91,932	-24.2		513,365	339,995	-33.8		176,312	102,357	-41.9		1,261,832	964,762	-23.5		689,914	525,784	-23.8
Treatment																			
Cervical excision (types 1. 2 and 3)	2,393	1,534	-35.9		5,069	3,213	-36.6		1,412	904	-36.0		10,386	6,981	-32.8		7,412	5,351	-27.8
Cancer surgery	4,835	4,318	-10.7		33,503	26,358	-21.3		9,863	8,945	-9.3		69,827	59,365	-15.0		42,356	36,226	-14.5
Chemotherapy	132,803	137,114	3.2		820,501	848,803	3.4		201,868	211,695	4.9		1,707,499	1,756,047	2.8		798,635	825,278	3.3
Radiotherapy^b^	4,049	3,725	-8.0		18,648	18,798	0.8		3,851	4,363	13.3		36,422	36,712	0.8		17,645	16,465	-6.7

Source: Brazilian National Health System Outpatient Information System (SIA/SUS); access in March 2021.

a) SUS: Brazilian National Health System; b) ‘Radiotherapy’ procedure data refer to June-December 2019 and June-December 2020.

 April and May 2020 were the most critical months for biopsy, which reached a 78.5% drop in May in the Northeast. Anatomical pathology tests, which include samples from biopsies and surgeries, fell most in May 2020 in the Northeast (-64.8%) and in June 2020 in the Midwest (-65.5%). Treatment of cervical cancer precursor lesions fell most in the Northeast, in April (-84.6%) and June 2020 (-71.7%). Cancer surgery dropped most in the Northeast, in May (-56.5%) and June (-36.8%). The largest drop in radiotherapy procedures was found in the Southern region in June (-18.7%). Compared to 2019, records of chemotherapy procedures remained stable in 2020 in all regions, and there was even a slight increase. We found that there was an upturn in all procedures and in all regions, especially with effect from August and September 2020.

The proportion of cervical and breast cancer histopathology tests performed in up to 30 days was higher in 2020 in virtually all the country’s five major regions, with the exception of the Northern region, where the proportion of breast cancer histopathology tests taking more than 60 days to be performed rose from 8.4% in 2019 to 29.1% in 2020.

## DISCUSSION

In Brazil, in 2020, almost all procedures related to cancer screening, diagnosis, and treatment suffered a decrease in relation to those recorded in 2019; except for chemotherapy, which maintained its production volume, with a slight increase in 2020. Screening exams suffered the greatest reductions, especially from April to June 2020. 

COVID-19 incidence rates, as well as the restriction measures adopted by state and local governments, varied between states and regions. However, a downward trend was seen in the performance of procedures, nationwide, and more sharply in April and May, with a pickup in production in the last quarter of 2020.

The results of this study indicated a considerable reduction in the performance of SUS cancer screening and diagnosis procedures in all regions of the country in the context of the COVID-19 pandemic, even though lockdown was not implemented as a nationwide measure and no centralized measures were adopted to suspend elective medical care. Despite population mobility restrictions, which may have led to a reduction in demand for routine outpatient care, health services and health professionals were overburdened.^16^

Considering only treatment procedures, those specifically for cervical cancer precursor lesions suffered more negative variation. In view of this finding, we have raised several hypotheses: (i) women diagnosed with precursor lesions might have chosen to wait for the pandemic to abate, before having treatment; (ii) the impact of overloading on outpatient services caused by meeting demands related to COVID-19; (iii) the suspension of elective procedures; and (iv) a decrease in precursor lesion diagnosis as a consequence of reduction in screening.

The time intervals between cervical and breast cancer screening tests being performed and diagnosis reports being released did not vary significantly. A reduction in diagnosis procedure time could be seen in some regions, indicating adherence to recommendations to prioritize symptomatic cases and diagnosis of people with altered screening test results during the pandemic.^10^ Another possibility with regard to reduction in total times lies in greater agility in the release of reports by laboratories and radiology clinics, due to the lower demand for elective procedures and the volume of tests performed in the period. We found a change in the time pattern for histopathology tests in the Northern region, with an increase in time during the pandemic. This is possibly a consequence of SISCAN only having been implemented in histopathology services in the state of Amazonas in 2020, making it difficult make a comparison between 2019 and 2020.^17^

 At the beginning of the pandemic, there was emphatic official guidance not to perform screening outside the current recommendations for age range and periodicity.^8^ Despite this, the data show that the pattern of lack of adherence to guidelines persisted in 2020,^18^ exposing those who were screened to greater risks, without any guarantee of the existence of benefits.

The effect of the pandemic on cancer individual care has been addressed in several international studies. A systematic review, published in 2021, identified 62 studies conducted in 15 countries, mostly in Europe and North America, related to delays and interruptions in the treatment of cancer people as a consequence of the pandemic: delays in treatment were reported by 77.5% of survey respondents, a 26.3% treatment interruption rate was identified in the longitudinal studies included in the review, as well as a 30.0% reduction in cancer-related hospitalizations.^19^ No studies were found that directly evaluated the effect of the pandemic on cancer screening and diagnosis, although there were estimates made by professionals involved in the management of some cancer screening programs in low- and middle-income countries.^20^

A study conducted in specialized oncology centers in 54 countries found that 88.0% reported difficulties in care during the pandemic; and 46.3% reported that more than 10.0% of individuals cared for had gone without a chemotherapy cycle.^21^

A study conducted in the United Kingdom using population-based modeling estimated between 3,291 and 3,621 additional deaths due to breast, oesophageal, lung and colorectal cancer over a five-year period as a result of delays in diagnosis of these cancers attributed to the pandemic.^22^

In the United States, a comparative study of Medicare data, that country’s national health insurance system, for the period March-July 2019 and March-July 2020, found a drop of 85.0% in breast cancer screening in April, the peak of the epidemic in the United States. Chemotherapy administration service revenues also showed a drop in April through June 2020, ranging from 9.6 to 31.2%.^23^

A study conducted in Brazil identified that average hospital admission rates for clinical cancer treatment decreased from 13.9 to 10.2 per 100,000 inhabitants between 2019 and 2020, representing a rate difference of -3.7/100,000 inhab. Admissions for surgical oncology treatment showed a decline of -5.8 per 100,000 inhabitants, with regional differences ranging from -2.2 to -10.8 per 100,000 inhabitants, and a more significant drop in the South and Southeast regions.^24^

Comparing procedures performed only between two years, 2019 and 2020, can be seen as a limitation of this study, considering that other factors could also contribute to the reduction in availability and use of services. However, in the monthly analysis, it can be seen that in the months prior to the pandemic (January and February), there were no significant drops in records, and there was even an increase in some cases.

Due to the change in 2019 in the way radiotherapy procedures are registered and funded,^15^ it was not possible to evaluate its variation between March and May, precisely the months when the other procedures presented the greatest variation in production. However, the small variation that occurred in the following months and its behavior similar to that of chemotherapy procedures indicate that radiotherapy was also affected less by the pandemic.

The cancer surgeries evaluated corresponded only to those recorded by hospitals with accredited oncology services. It is possible that cancer surgeries performed in general hospitals suffered the greatest effect of the pandemic, due to overloading of these services and hospital bed occupation. As there is not a specific code for cancer surgery performed in hospitals that are not accredited, it was not possible to carry out this analysis in this study.

The results presented here, in agreement with those from other countries, indicate that cancer control actions may have suffered from the effects of the COVID-19 pandemic, especially in the first months after the beginning of the community transmission of the disease.^21^^,^^23^ In Brazil, cancer screening and diagnosis were more affected than treatment, a result to be expected considering the recommendations in force and the balance between risks and benefits of maintaining screening actions in an unfavorable epidemiological scenario such as that of COVID-19.

One year after the start of the pandemic, designing strategies to mitigate harm due to possible delays resulting from the presence of COVID-19 is critical. Among these strategies, the following initiatives, included in the survey conducted by the International Agency for Research on Cancer, are worth mentioning: (i) development of applications or specific telephone numbers for scheduling oncology appointments and clarifying doubts; (ii) screening test reports made available online; (iii) teleconsultation for individuals with positive tests; (iv) free transportation for individuals with positive screening tests; and (v) the engagement of young volunteers who identify and support individuals in need and with difficulties in access to oncology care.^20^

Despite the efforts already begun in several countries, a systematic review to evaluate the impact of measures adopted to reduce the effects of COVID-19 on cancer care, including postponement or change in therapeutic regimen, changes in screening intervals and protective measures in treatment centers (isolated wards for people with COVID-19, teleconsultations), did not identify consistent effects, given the lack of standardization in evaluation methods, reiterating the importance of measuring and documenting the strategies adopted.^25^

In the period prior to the pandemic, Brazil already had difficulties in organizing screening and access to diagnostic procedures, as well as long waiting times for cancer treatment.^26^^-^^28^ Once the pandemic is under control, it will be necessary to concentrate efforts on implementing a prioritization strategy, based on risk stratification: diagnostic confirmation and treatment of symptomatic suspected cancer cases, and of people with positive screening tests, before or during the pandemic; and active tracing of members of the target population who have never screened or whose screening is overdue according to recommended periodicity.^9^

Other relevant aspects for planning actions related to cancer control after the COVID-19 pandemic has been controlled include the performance of cancer screening tests, in the appropriate age group and periodicity, and agility in the diagnosis and treatment of confirmed cases. Although this study did not find variations in the records of procedures by place of residence, in a country with continental dimensions such as Brazil, it is essential to reevaluate the access and transportation conditions of people who live in isolated areas or very distant from diagnosis and treatment centers, especially in a context of people’s reduced movement and mobility.

Despite the devastating effects of the COVID-19 pandemic, this can be a time for planning, in the sense of providing an opportunity to reorganize the oncology care network and early cancer detection programs. It is necessary to evolve from the opportunistic screening model, in which tests are performed on demand or requested by health professionals, to the organized model, in which the target population is invited to undergo tests with the recommended periodicity. In this way, it will be possible to establish centralized control of screening actions, a fundamental strategy for greater effectiveness of cancer control actions in Brazil.^29^
